# Colorectal cancers with a residual adenoma component: Clinicopathologic features and *KRAS* mutation

**DOI:** 10.1371/journal.pone.0273723

**Published:** 2022-09-09

**Authors:** Hyoun Wook Lee, Boram Song, Kyungneun Kim

**Affiliations:** 1 Department of Pathology, Samsung Changwon Hospital, Sungkyunkwan University School of Medicine, Changwon, Republic of Korea; 2 Department of Pathology, Kangbuk Samsung Hospital, Sungkyunkwan University School of Medicine, Seoul, Republic of Korea; National Hospital Organization Minami Wakayama Medical Center, JAPAN

## Abstract

**Background/Aim:**

Colorectal cancer is well known for its “adenoma-carcinoma” sequential carcinogenesis. Some colorectal cancers demonstrate a residual adenoma component during progression from adenoma to invasive carcinoma. However, the clinicopathological significance of residual adenoma component remains unclear. In this study, we aimed to investigate the clinicopathologic and molecular characteristics including the *KRAS* mutation in colorectal cancers containing a residual adenoma component.

**Materials and methods:**

In this study, 498 surgically resected colorectal cancer patients were enrolled. Their detailed clinicopathologic features and results of molecular study including *KRAS* mutation test and microsatellite instability were analyzed.

**Results:**

A residual adenoma component was identified in 42 (8.4%) patients with colorectal cancer. The presence of a residual adenoma component was associated with a high frequency of the *KRAS* mutation (65%, p = 0.031) as well as indolent clinicopathological features, including polypoid gross type (p < 0.001), well-differentiated histology (p < 0.001), low pT (p < 0.001) and pN stage (p = 0.003), absence of vascular invasion (p = 0.005), and a better progression-free prognosis (p = 0.029). The cases with an adenoma component had a 35.7% discordance rate on the *KRAS* mutation tests in their adenoma and carcinoma regions.

**Conclusion:**

In conclusion, colorectal cancer with a residual adenoma component showed indolent clinicopathologic features and frequent *KRAS* mutations. Due to the discordance in the incidence of the *KRAS* mutation between the adenoma and carcinoma components, the adenoma component should be documented in the pathology report, and care should be taken not to include the adenoma component when collecting samples for molecular testing.

## Introduction

Colorectal cancer is a representative neoplasm in which the sequential carcinogenesis of adenoma-carcinoma progression is well known [1, 2]. During the adenoma-carcinoma sequence, an adenoma progresses to a carcinoma due to accumulation of a number of genetic mutations, such as those of APC, *KRAS*, and TP53 [3, 4]. As the colorectal cancer grows in size, the adenoma portion may either disappear or remain a part of the tumor. A residual adenoma component in colorectal cancer has been noted due to the discordance of molecular test results in this component with that of an invasive component, but few reports have described the clinicopathologic features of colorectal cancers that include residual adenoma components [5–10]. Special subtypes of colorectal cancer with extremely well-differentiated (WD) histology, such as adenoma-like adenocarcinoma or villous adenocarcinoma, have also been reported; these lesions have distinctive clinical features and a better prognosis than conventional adenocarcinoma along with the possibility of being mistaken for an adenoma during biopsy [11–13]. However, the residual adenoma component in colorectal cancers histologically differs from both conventional adenocarcinoma and these special subtypes with WD histology. There have been few studies on the characteristics of colorectal cancer including the residual adenoma component. Druliner *et al*. reported that colorectal cancer with residual polyp of origin was frequently found at lower grade or stage, but showed the same prognosis as conventional adenocarcinoma within the same stage [14]. Also, there was no molecular difference according to the presence or absence of residual polyp of origin in enrolled colorectal cancers [14].

The epidermal growth factor receptor (EGFR) is a major therapeutic target in colorectal cancers, and the *KRAS* mutation test is essential in clinical practice for predicting resistance to EGFR inhibitors during metastatic colorectal cancer treatment [14–16]. The *KRAS* mutation is a relatively early event in colorectal cancer carcinogenesis because this mutation has been identified in adenomas and even in non-neoplastic hyperplastic conditions [4, 6, 17, 18]. The incidence of *KRAS* mutation was reported to range from 13.6–35.0% and from 30–50% in adenomas and carcinomas, respectively [19–27]. According to the previous report by Hershkovitz *et al*., the discordance rate between the adenoma and invasive carcinoma components in the same tumor was 23% and they suggested that adenoma components should not be included when collecting tissue samples from colorectal cancers that contain an adenoma component [6].

Studies on colorectal adenocarcinoma with a residual adenoma component have focused on molecular differences between adenoma and invasive components, and studies on the clinicopathologic correlation of colorectal cancer with an adenoma component are rare. Therefore, this study aimed to investigate both the clinicopathologic and molecular characteristics of colorectal cancers containing residual adenoma components.

## Materials and methods

### Patients and clinical data

This retrospective study was approved by the Institutional Review Board of Kangbuk Samsung Hospital, Seoul, South Korea (IRB No.2019-07-022), which waived the requirement for informed consent due to the use of de-identified data and specimens remaining after the end of treatment. Initially, we included 550 consecutive colorectal cancer specimens resected at Kangbuk Samsung Hospital (Seoul, South Korea) between January 2006 and December 2014. Fifty-two patients who underwent pre-operative chemoradiation therapy were excluded and 498 colorectal cancer patients were finally enrolled in this study. Clinical information, including survival time, was obtained from the patients’ electronic medical records.

### Gross examination and microscopic evaluation of resected colorectal cancer specimens

The gross characteristics of the tumor, including tumor location and gross type, were recorded. Two pathologists reviewed independently all glass slides and recorded microscopic features including histological classifications according to the 2010 World Health Organization Tumor Classification [28], tumor stage according to the 2010 AJCC Tumor Node Metastasis Staging System [29]. Presence of a residual adenoma component along with other histologic features, including lymphovascular invasion or perineural invasion, were recorded after microscopic review.

### DNA extraction from colorectal cancer tissue

After microscopic review, the areas that contained the adenoma and carcinoma components were labelled on their respective glass slides. A paraffin block was sectioned into slices 5 μm in thickness, and the marked adenoma and carcinoma areas were collected separately. Deparaffinization was performed with xylene, and then the tissue was washed in 100% ethanol. Genomic DNA was isolated using conventional methods with a QIAamp DNA Kit (Qiagen, Courtaboeuf, France) according to the manufacturer’s instructions.

### Polymerase chain reaction for KRAS

A PNA Clamp *KRAS* Mutation Detection Kit (Panagene Inc., Daejeon, Korea) was used to detect *KRAS* mutations in codons 12 and 13 with real-time polymerase chain reaction (PCR) according to the manufacturer’s instructions. Real-time PCR reactions were performed on a CFX96 PCR detection system (Bio-rad, Philadelphia, PA, USA). The efficiency of PCR clamping was determined by measuring the threshold cycle (Ct) values. Mutation status was determined based on a Ct value difference < 2 between the control and sample.

### Microsatellite instability test

Microsatellite instability (MSI) was analyzed by PCR amplification using fluorescent dye-labeled primers for five dinucleotide markers (BAT-26, BAT-25, D5S346, D2S123, and D17S250) that are specific for microsatellite loci. MSI was defined as a band shift in either of the two alleles or as the appearance of a different sized band in the tumor sample. Tumors that showed instability in ≥ 2/5 of these tested markers were classified as having a high degree of MSI; instability in 1/5 of markers was classified as a low MSI. The status of MSI-low (MSI-L) was assigned if at least one but not all of the loci showed instability, whereas a microsatellite stable (MSS or MSI-S) status was granted if all loci were stable. Only high MSI cases were considered MSI positive.

### Statistical analysis

Data were analyzed using PASW Statistics 18 (SPSS, Inc., Chicago, IL, USA) software. Statistical significance in discriminating between presence and absence of a residual adenoma component was determined by crosstabs, Pearson’s chi-square test, and Fisher’s exact test. For survival analysis, Kaplan-Meier survival analysis and logistic regression test were used. To obtain the hazard ratios, Cox regression tests were performed. Differences were regarded as statistically significant at p < 0.05.

## Results

**Fig 1:**
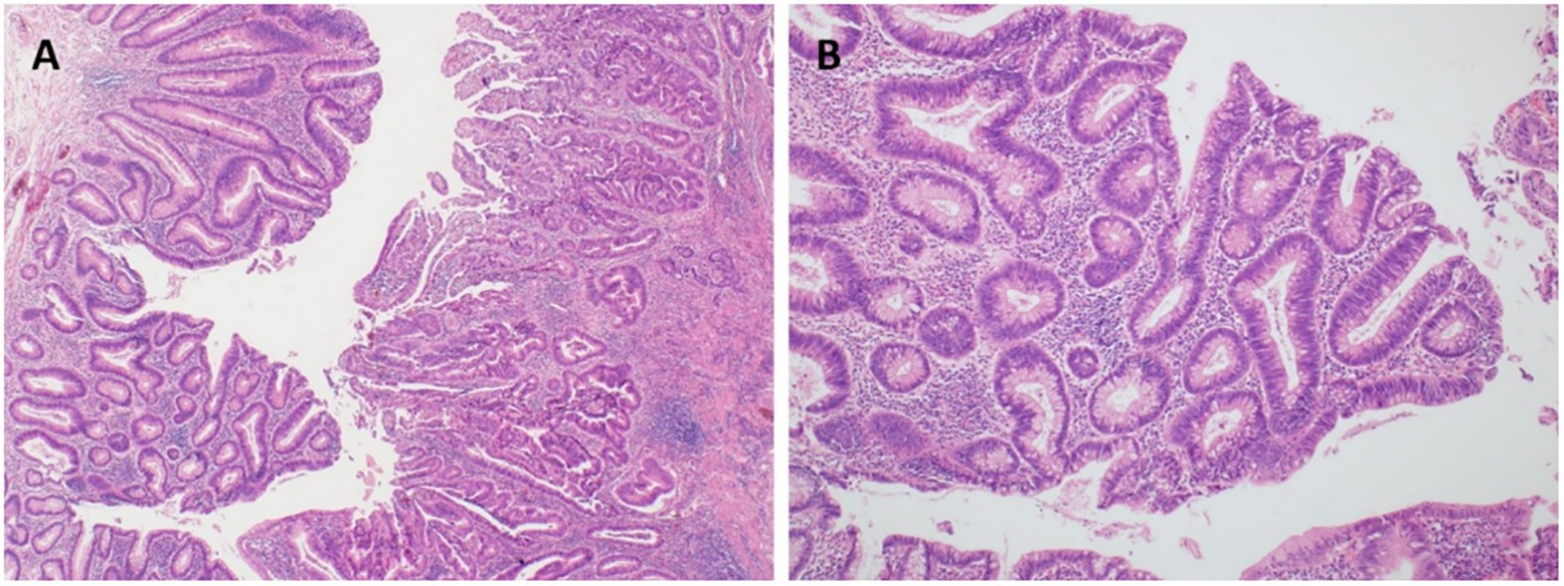
The representative microscopic features of colorectal cancer with a residual adenoma component. The remaining adenoma component is present on the left side of the invasive adenocarcinoma component (A). The adenoma component shows low-grade dysplasia without invasive epithelial islands or desmoplastic stroma (B).

The clinicopathological data for all 498 cases are shown in Table 1. The patients were 298 (59.8%) males and 200 (40.2%) females, and their median age was 63 years (range: 26–90 years). There were 42 (8.4%) patients with tumors that contained a residual adenoma component; representative figures of these patients are shown in Fig 1. The histologic type of adenoma was tubular in all cases. Among the 293 cases who received a *KRAS* mutation test, this mutation was identified in 37.9% and was located on codon 12 and 13 at a rate of 30.7% and 7.2%, respectively. There were no cases in which mutations were found on two codons at the same time.

**Table 1:** Clinicopathologic features of 498 colorectal cancer cases.

Variables	Subgroups	Number of cases (%)
Tumor location		
	right colon	124 (24.9)
	left colon	162 (32.5)
	rectum	213 (42.8)
Gross type*		
	polypoid	56 (19.1)
	ulcerofungating	129 (44.0)
	ulceroinfiltrative	100 (34.1)
	infiltrative	8 (2.7)
Differentiation		
	well	33 (6.6)
	moderate	411 (82.5)
	poorly	42 (8.4)
	mucinous	12 (2.4)
pT stage		
	1	39 (7.8)
	2	66 (13.3)
	3	341 (68.5)
	4	52 (10.4)
pN stage		
	0	248 (49.8)
	1	161 (32.3)
	2	89 (17.9)
Lymphatic invasion		
	absent	300 (60.2)
	present	198 (39.8)
Vascular invasion		
	absent	437 (87.8)
	present	61 (12.2)
Perineural invasion		
	absent	371 (74.5)
	present	127 (25.5)
Adenoma component		
	absent	456 (91.6)
	present	42 (8.4)
*KRAS* *		
	wild	182 (62.1)
	mutation, codon 12	90 (30.7)
	mutation, codon 13	21 (7.2)
Microsatellite instability**		
	stable/ low	249 (90.5)
	high	26 (9.5)

*Gross type and *KRAS* mutation result were evaluable in 293 cases.

** Microsatellite instability was tested in only 275 cases.

**Fig 2:**
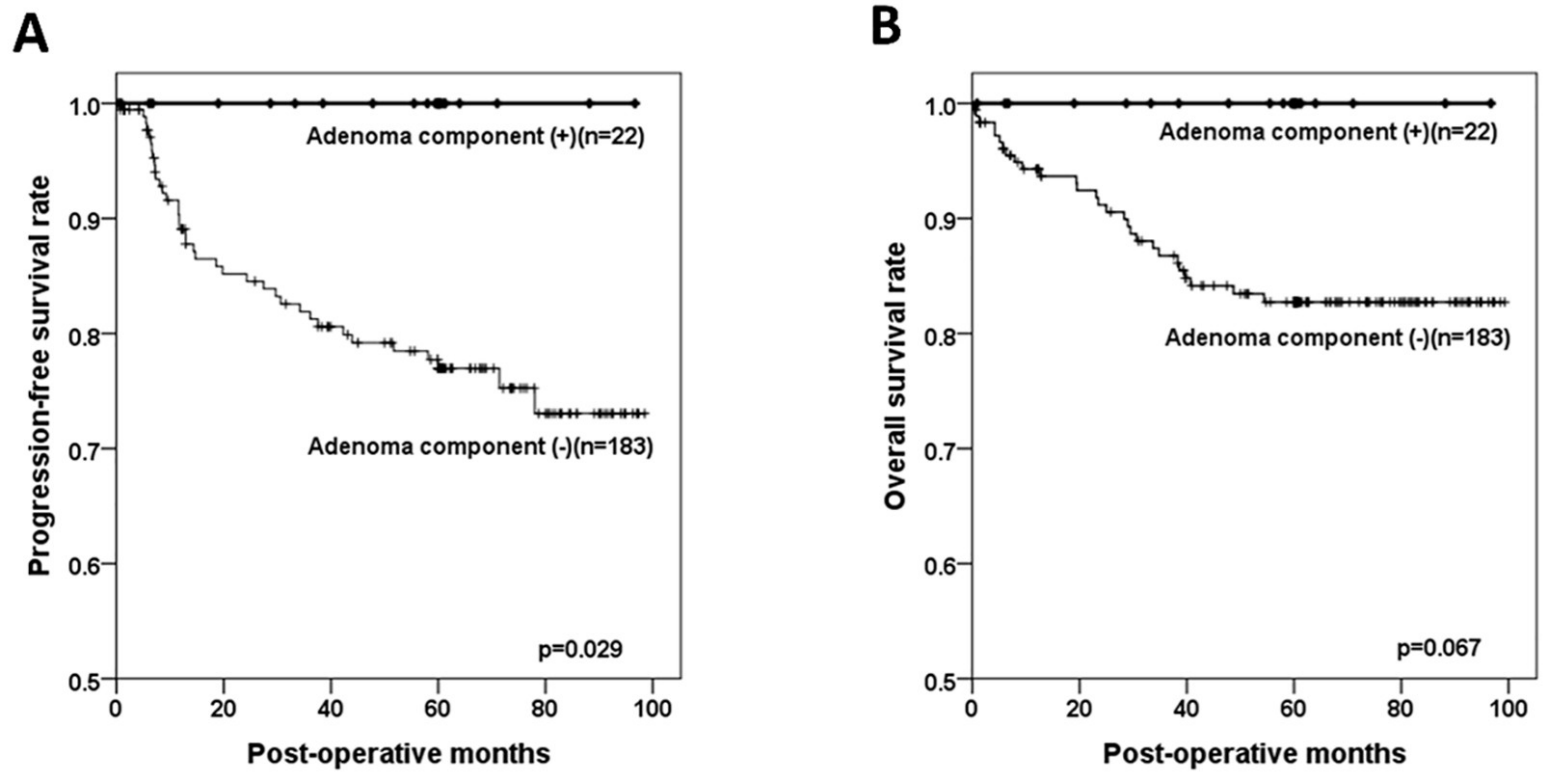
The Kaplan-Meier survival curves based upon the presence of a residual adenoma component. Patients with colorectal cancers that contained an adenoma component showed remarkably better progression-free (A) and overall (B) survival than those with no such component.

The presence of a residual adenoma component was associated with polypoid gross type (p < 0.001), WD histology (p < 0.001), low pT (p < 0.001) and pN stage (p = 0.003), and absence of vascular invasion (p = 0.005; Table 2). The mean patient age (65.0 years) in cases with an adenoma component was slightly higher than that found in cases with no such remnant (63.0 years; p = 0.031). The sex, tumor location, and frequency of lymphatic and perineural invasion did not show significant association with presence of an adenoma component. In addition, the mean values of tumor size were 4.6 and 4.9 cm in patients with and without an adenoma component, respectively, and the difference was not significant (p = 0.080). The survival analysis revealed that cases with a remnant adenoma component showed significantly better progression-free survival (p = 0.029), and no patients experienced local recurrence or distant metastasis of colorectal cancer during a mean of 49.9 months of follow-up (Fig 2A). Overall survival rate showed a similar tendency, but the difference was not statistically significant (p = 0.067; Fig 2B). A Cox regression test confirmed that the presence of a residual adenoma component was not a significant factor of progression-free survival, although the tumor differentiation, and pT and pN stage were significant (Table 3).

**Table 2:** Clinicopathologic characteristics of colorectal cancer with adenoma component.

Variables	Subgroups	Adenoma component		
		present	absent	p-value
Sex				0.744
	male	24 (57.1)	274 (60.1)	
	female	18 (42.9)	182 (39.9)	
Tumor location				0.951
	right	11 (26.2)	113 (24.8)	
	left	14 (33.3)	147 (32.2)	
	rectum	17 (40.5)	196 (43.0)	
Gross type*				<0.001
	polypoid	12 (60.0)	44 (16.1)	
	ulcerofungating	7 (35.0)	122 (44.7)	
	ulceroinviltrative	1 (5.0)	99 (36.3)	
	infiltrative	0 (0)	8 (2.9)	
Differentiation				<0.001
	well	22 (52.4)	11 (2.4)	
	moderate	17 (40.5)	394 (86.4)	
	poorly	1 (2.4)	41 (9.0)	
	mucinous	2 (4.8)	10 (2.2)	
pT				<0.001
	1	16 (38.1)	23 (5.0)	
	2	10 (23.8)	56 (12.3)	
	3	15 (35.7)	326 (71.5)	
	4	1 (2.4)	51 (11.2)	
pN				0.003
	0	31 (73.8)	217 (47.6)	
	1	9 (21.4)	152 (33.3)	
	2	2 (4.8)	87 (19.1)	
Lymphatic invasion				0.082
	absent	30 (71.4)	270 (59.2)	
	present	12 (28.6)	186 (40.8)	
Vascular invasion				0.005
	absent	42 (100.0)	395 (86.6)	
	present	0 (0)	61 (13.4)	
Perineural invasion				0.096
	absent	36 (85.7)	335 (73.5)	
	present	6 (14.3)	121 (26.5)	

*Gross type was evaluable in 293 cases.

**Table 3:** Univariate and multivariate analyses for prediction factors for disease-free survival.

Variables	Univariate		Multivariate	
	p-value	Hazard ratio (95% CI)	p-value	Hazard ratio (95% CI)
Tumor differentiation (WD and MD vs. PD)	<0.001	13.508 (6.846–26.650)	<0.001	9.458 (4.674–19.140)
pT stage (1 and 2 vs. 3 and 4)	0.017	11.261 (1.545–82.104)	0.126	4.800 (0.643–35.812)
pN stage (0 vs. 1 and 2)	<0.001	4.274 (2.022–9.035)	0.031	2.350 (1.081–5.111)
Residual adenoma component (present vs. absent)	0.160	24.12 (0.284–2051.846)		

WD, well-differentiated; MD, moderately differentiated; PD, poorly differentiated

According to molecular tests, presence of a residual adenoma component was correlated with a high frequency of the *KRAS* mutation (65%, p = 0.031) but not with MSI (Table 4). When separate *KRAS* mutation tests were performed in the regions with a remnant adenoma component, the *KRAS* mutation was confirmed in 9 cases (64.3%): 8 at codon 12 and 1 at codon 13 (Table 5). Discordance in the *KRAS* mutation results between the adenoma and carcinoma areas was identified in 5 of 14 cases (35.7%). Three cases with a wild-type *KRAS* mutation in the adenoma component housed a *KRAS* mutation in the carcinoma component, and two cases with codon 12 mutation in the residual adenoma component demonstrated a wild-type *KRAS* mutation in the carcinoma component.

**Table 4:** The results of molecular tests according to presence of adenoma component.

Variables	Subgroups	Adenoma component		
		present	absent	p-value
*KRAS* *				0.031
	wild	7 (35.0)	175 (64.1)	
	mutation, codon 12	10 (50.0)	80 (29.3)	
	mutation, codon 13	3 (15.0)	18 (6.6)	
Microsatellite instability**				>0.999
	stable/ low	18 (80.0)	231 (90.6)	
	high	2 (10.0)	24 (9.4)	

*The *KRAS* mutation test was evaluable in 293 cases.

**Microsatellite instability test was evaluable in 275 cases.

**Table 5:** *KRAS* mutation in adenoma and carcinoma components and clinicopathologic features.

Patient number	*KRAS* mutation		pT	pN	Differentiation	Gross type	Size (cm)
	adenoma component	carcinoma component					
1	wild	wild	3	0	well	ulcerofungating	7.5
2	wild	wild	3	0	moderate	polypoid	9.0
3	wild	codon 12	2	0	well	polypoid	2.5
4	wild	codon 12	1	0	well	ulcerofungating	4.6
5	wild	codon 13	3	0	well	ulcerofungating	8.0
6	codon 12	codon 12	1	0	well	polypoid	2.0
7	codon 12	codon 12	3	1	mucinous	ulcerofungating	8.8
8	codon 12	codon 12	3	1	well	ulceroinfiltrative	2.5
9	codon 12	codon 12	2	0	well	polypoid	3.2
10	codon 12	codon 12	0	0	well	polypoid	3.0
11	codon 12	codon 12	1	0	well	polypoid	5.0
12	codon 12	wild	2	1	well	polypoid	2.7
13	codon 12	wild	1	1	well	polypoid	2.0
14	codon 13	codon 13	2	0	well	ulcerofungating	10.5

## Discussion

In this study, we evaluated the frequency of a residual adenoma component in colorectal cancers and their clinicopathologic features. The cases that had a residual adenoma component demonstrated older patient age, better histologic differentiation, more frequent polypoid gross appearance, lower pT and pN stage, less frequent vascular invasion, and better progression-free survival. In addition, *KRAS* mutations were more frequently identified in colorectal cancers that contained an adenoma component.

Few studies have characterized the clinicopathologic features of colorectal cancers containing a residual adenoma component. Druliner *et al*. conducted a large-scale study involving 4,647 colorectal cancers, and residual adenoma components were found in 11.7% of them [14]. They reported that colorectal cancer with residual polyp of origin was frequently found at lower grades or stages, but within the same stage, the prognosis was the same as that of conventional adenocarcinoma, and there was no difference according to the presence of residual polyp of origin in molecular examination [14]. However, the molecular study was conducted with only 10 cases and the detailed results of *KRAS* mutation were not shown. Extremely WD adenocarcinomas, such as adenoma-like adenocarcinoma or villous adenocarcinoma, have also been reported [11–13]. According to a report by Loy *et al*., villous adenocarcinoma was identified in 8.6% of all resected colorectal adenocarcinomas, and more than half were not accompanied by severe atypia, which made it difficult to diagnose adenocarcinoma on biopsy, and the patients showed a good prognosis [11]. Subsequently, Gonzalez *et al*. reported an extremely WD adenocarcinoma subtype termed an “adenoma-like adenocarcinoma,” which had the following characteristics: 1) a lower N stage compared to the same T stage, 2) a better prognosis than conventional adenocarcinoma, 3) difficulty in distinguishing the adenoma from the carcinoma on biopsy specimens, and 4) frequent *KRAS* mutation (58%) [13]. In another recently published report, the adenoma-like adenocarcinoma subtype showed similar clinicopathologic characteristics: lower pN and pT stage and good recurrence-free survival rate compared to conventional adenocarcinoma subtype [13]. These extremely WD adenocarcinoma subtypes described in above previous reports could therefore be confused with a residual adenoma component in this study. However, the remnant adenoma components included in this study were clearly distinguishable from the invasive components, and no further evidence of invasion, such as “epithelial islands in desmoplastic stroma” as reported by Loy *et al*., was identified in our cases [11]. All 42 cases of adenocarcinoma with a residual adenoma component included in this study were diagnosed as adenocarcinoma in preoperative biopsy without diagnostic confusion with adenoma. However, if only the adenoma component is obtained from the preoperative biopsy, and it is difficult to confirm the pathological diagnosis of colorectal adenocarcinoma, the endoscopic or radiologic findings could be helpful in decision of repeated biopsy.

*KRAS* mutations are known to be involved in the early stages of colon cancer carcinogenesis [4, 18, 19, 21, 22, 30]. In particular, the *KRAS* mutation is associated with histologic progression of adenomas toward a villous histology and higher grades of dysplasia, which suggests that this mutation occurs during the late stages of adenoma progression along with an advanced morphological phenotype and not as an intermediary step in development from adenoma to carcinoma [21, 31–33]. Discordance in the presence of a *KRAS* mutation in carcinoma and adenoma components was initially reported by Bos *et al*. [34]. Later, Hershkovitz *et al*. found a 23% discordance rate in 70 colorectal adenocarcinomas that contained an adenoma component [6]. Although we tested for *KRAS* mutation discordancy in only 14 cases, we confirmed discordant results in 5 (35.7%) of these cases (Table 5). The *KRAS* mutation test is one of the main tests to determine whether the EGFR pathway is functioning properly when anti-EGFR agents are prescribed in patients with metastatic colorectal cancer. Therefore, in pathologic diagnosis of colorectal cancers, any residual adenoma component should be examined and recorded in pathology report. In addition, when conducting a *KRAS* mutation test in patients with colorectal cancer that contains an adenoma component, only the invasive carcinoma component should be harvested to ensure accurate test results.

Our study had a few limitations. First, because this study included only resected colorectal cancer cases, we were unable to evaluate the early colorectal cancer arising in polyps that usually removed by endoscopic resection. Further studies including early colorectal cancer or adenocarcinoma arising in colon polyps with a higher proportion of residual adenoma components will be needed. Second, this study was performed with single-center cases in Korea. Further studies are needed to apply the features of colorectal cancer with residual adenoma components to other races and countries. Lastly, this study only included the results for *KRAS* mutation of codon 12 and 13 and MSI molecular markers, and these tests were not performed in all cases. Further evaluation of the recommended molecular biomarkers for colorectal cancers, including more number of codon of *KRAS*, NRAS and BRAF, may also be required [33, 35].

In conclusion, colorectal cancer with a residual adenoma component had a low incidence of 8.4%. Colorectal cancer with a residual adenoma component showed indolent clinical characteristics including WD histology, low pT and pN stage, low incidence of vascular invasion and better progression-free survival rate. In a molecular aspect, cases with a residual adenoma component revealed frequent *KRAS* mutation (65%) and the discordance of *KRAS* mutation test between adenoma and invasive carcinoma area was found in 35.7%. Therefore, the residual adenoma component should be recorded in pathology report and carefully excluded when collecting tissue samples for the *KRAS* mutation test.

## Supporting information

S1 Data (PDF)
