# Correction: Colorectal cancers with a residual adenoma component: Clinicopathologic features and *KRAS* mutation

**DOI:** 10.1371/journal.pone.0334953

**Published:** 2025-10-17

**Authors:** Hyoun Wook Lee, Boram Song, Kyungeun Kim

The third author’s name is spelled incorrectly. The correct name is: Kyungeun Kim. The correct citation is: Lee HW, Song B, Kim K (2022) Colorectal cancers with a residual adenoma component: Clinicopathologic features and *KRAS* mutation. PLoS ONE 17(9): e0273723. https://doi.org/10.1371/journal.pone.0273723.
