# Supplementary material for: Colorectal cancers with a residual adenoma component: Clinicopathologic features and KRAS mutation
Source: PLoS One. 2022 Sep 9;17(9):e0273723. doi: 10.1371/journal.pone.0273723 (PMC9462729; doi:10.1371/journal.pone.0273723)
Supplement: S1 Data — (PDF) [file pone.0273723.s001.pdf]

case_no	age	sex	diff	loc_R_L_R	size	lym_inv	vas_inv
237	69	1	2	3	6	0	0
235	74	1	4	3	8	0	0
229	54	1	4	3	4.5	0	0
280	59	1	4	3	3	0	0
211	51	1	4	3	3	0	0
189	79	2	3	3	8	1	0
123	63	2	3	3	11	1	0
308	50	1	3	3	7	1	1
188	66	1	3	3	5.6	1	0
185	79	1	3	3	5	1	0
267	67	2	3	3	4.7	1	1
243	46	1	3	3	7.7	1	1
95	47	1	3	3	7.5	1	1
297	77	1	2	3	5	1	1
255	62	1	2	3	8.5	0	0
208	71	1	2	3	5.6	1	1
221	65	1	2	3	4.5	0	0
290	78	1	2	3	7	0	0
293	80	1	2	3	7	1	0
198	38	1	2	3	6.5	0	0
124	87	2	2	3	4.5	1	0
210	49	1	2	3	7.5	0	0
103	79	2	2	3	3.5	0	0
306	46	2	2	3	5.5	1	1
204	71	1	2	3	6.5	0	0
142	61	1	2	3	7	0	0
242	74	2	2	3	3.5	0	0
310	49	1	2	3	6	1	0
265	74	2	2	3	6	1	0
137	76	1	2	3	5.8	1	0
241	47	1	2	3	6.5	0	0
216	71	1	2	3	4	1	0
226	64	1	2	3	6	0	0
220	70	2	2	3	5	0	0
253	55	1	2	3	6	0	0
186	46	1	2	3	10.5	0	0
120	57	2	2	3	4	1	0
160	59	2	2	3	4	1	0
251	55	2	2	3	6.2	0	1
263	46	1	2	3	5	0	1
324	63	1	2	3	4.5	0	0
316	70	1	2	3	4	1	0

154	81	2	2	3	7	1	0
133	59	1	2	3	5	0	0
292	73	1	2	3	6.5	1	0
315	60	1	2	3	7	1	0
203	55	2	2	3	4.5	0	0
94	46	1	2	3	10	0	0
318	71	2	2	3	4.2	0	0
268	56	2	2	3	5	0	0
269	61	1	2	3	4	1	1
254	70	1	2	3	6.5	0	0
228	58	1	2	3	4.2	1	1
172	63	1	2	3	7.5	0	0
233	79	1	2	3	4.5	0	0
183	64	2	2	3	5.2	1	1
167	67	1	2	3	5.3	0	0
115	67	2	2	3	5.5	0	0
155	59	2	2	3	5.5	1	0
117	49	1	2	3	11	1	0
96	62	1	2	3	7.5	0	0
244	60	1	2	3	4	0	0
141	55	1	2	3	5	1	0
158	66	2	2	3	4.7	0	0
176	65	2	2	3	3	1	0
148	57	2	2	3	6.8	0	0
301	68	1	2	3	4.5	0	0
99	81	2	2	3	5.8	0	0
130	71	1	2	3	3	1	0
266	48	1	2	3	5	1	0
270	65	1	2	3	8.5	0	0
219	58	2	2	3	4	0	0
206	59	1	2	3	3.5	1	0
165	66	2	2	3	4.2	0	0
250	70	1	2	3	3.8	0	0
212	67	1	2	3	2.4	0	0
262	67	1	2	3	4.1	1	0
101	58	1	2	3	2	0	0
277	58	1	2	3	4.5	0	0
275	69	1	2	3	9.5	1	0
205	59	2	2	3	2.2	0	0
144	68	2	2	3	3	0	0
287	56	2	2	3	4.6	0	0
227	64	1	2	3	4.5	0	0
149	52	2	2	3	6	0	0

201	44	2	2	3	4	0	0
153	52	1	2	3	4.5	1	1
196	60	1	2	3	2.2	0	0
271	48	1	2	3	3	1	0
279	66	1	2	3	2.5	0	0
200	67	1	2	3	2	0	0
181	41	1	2	3	2.6	0	0
169	56	2	2	3	9	0	0
245	73	1	1	3	4.5	0	0
257	63	2	1	3	7	0	1
145	72	1	2	3	8	0	0
132	59	2	2	3	5	1	0
104	57	1	2	3	5	0	0
273	65	1	2	3	3	0	0
197	57	2	2	3	1.8	0	0
10	60	1	2	3	1.8	1	0
121	59	2	2	3	4.3	0	0
146	69	2	1	3	11	0	0
202	49	2	1	3	4.2	0	0
163	72	1	1	3	3.3	0	0
127	71	1	1	3	3	0	0
499	78	1	3	3	8	1	0
494	71	1	3	3	4.1	0	0
655	61	1	3	3	7.1	1	0
646	83	2	3	3	6.3	1	0
463	74	1	3	3	4.7	0	0
544	30	1	2	3	6	1	0
621	53	2	2	3	5.2	1	0
497	47	2	2	3	5	0	0
529	65	1	2	3	2.5	0	0
493	75	1	2	3	2.2	0	0
459	72	2	2	3		1	1
475	54	1	2	3	4.6	1	1
553	87	1	2	3	6	1	1
545	72	2	2	3	3.5	1	0
608	62	2	2	3	8	1	0
617	72	1	2	3	6.5	1	0
625	57	1	2	3	5.2	1	0
664	52	2	2	3	2.3	1	0
416	47	1	2	3	5.3	0	0
548	77	1	2	3	6	0	0
669	72	2	2	3	5.9	0	1
466	60	2	2	3	2.7	0	0

468	72	1	2	3	6	0	0
461	72	1	2	3	7	1	0
534	65	1	2	3	2	1	1
570	78	1	2	3	5.5	1	0
581	48	1	2	3	2.8	1	0
436	49	1	2	3	5	1	0
445	63	1	2	3	8.8	1	0
454	53	1	2	3	4.3	1	0
511	51	2	2	3	3	1	0
566	63	1	2	3	5.3	1	0
568	57	2	2	3	4	1	0
687	73	2	2	3	2.5	1	0
532	53	1	2	3	8	0	0
562	54	1	2	3	6	0	0
693	57	1	2	3	2.4	0	0
405	65	1	2	3	3.5	0	0
409	80	2	2	3	8.2	0	0
492	58	1	2	3	0.5	0	0
518	82	1	2	3	4.5	0	0
547	55	1	2	3	6	0	0
579	69	1	2	3	5	0	0
598	54	2	2	3	2.5	0	0
424	74	1	2	3	5	1	0
457	66	1	2	3	5.5	1	1
586	53	1	2	3	5	1	0
574	79	1	2	3	4.5	1	0
640	60	1	2	3	4.7	1	0
433	72	1	2	3	7	0	0
546	68	1	2	3	5	0	0
572	58	1	2	3	2.5	0	0
410	82	2	2	3	5.5	0	0
437	66	1	2	3	8.5	0	0
439	65	1	2	3	5.5	0	0
444	74	2	2	3	4.2	0	0
450	73	2	2	3	8	0	0
460	55	2	2	3	2.7	0	0
471	67	1	2	3	5	0	0
487	63	2	2	3	2.3	0	1
495	50	1	2	3	2	0	0
496	51	1	2	3	3	0	0
519	26	1	2	3	4.3	0	0
525	83	2	2	3	5	0	0
571	72	1	2	3	3	0	0

602	67	1	2	3	4.6	0	0
609	77	2	2	3	6	0	0
634	71	1	2	3	3.2	0	0
642	73	2	2	3	7	0	0
682	46	2	2	3	3.9	0	0
512	69	1	2	3	3	1	1
484	76	2	2	3	4	1	0
656	39	1	2	3	2.5	1	0
406	58	1	2	3	2.5	0	0
589	45	2	2	3	2.7	0	0
654	60	1	2	3	2.3	0	0
524	59	2	2	3	4.9	1	0
692	86	1	2	3	5.5	1	0
430	64	1	2	3	3	0	0
434	66	2	2	3	2.5	0	0
472	42	1	2	3	1.5	0	0
530	74	2	2	3	2	0	0
552	45	1	2	3	5	0	0
627	76	2	2	3	3.3	0	0
665	77	2	2	3	2.5	0	0
666	61	2	2	3	3	0	0
588	60	1	2	3	2	1	0
652	56	2	2	3	0.6	1	0
585	63	1	2	3		0	0
615	71	1	2	3	1.5	0	0
541	67	2	2	3	2.5	1	0
413	56	1	2	3	1.4	0	0
420	78	1	2	3	1.6	0	0
441	43	1	2	3	1.5	0	1
521	52	2	2	3	2	0	0
593	73	2	2	3	2.1	0	0
594	66	1	2	3	1.7	0	0
447	48	1	1	3	8.5	0	0
476	76	1	1	3	2.5	0	0
486	50	1	1	3	11	0	0
607	46	1	1	3	3.5	1	0
576	73	1	1	3	2.7	1	0
543	31	1	1	3	2.5	1	0
650	54	1	1	3	10.5	0	0
417	61	2	1	3	2	1	0
404	72	1	1	3	3	0	0
520	78	2	1	3	4.6	0	0
106	47	1	4	2	3	0	0

114	38	2	3	2	3.5	1	1
259	50	1	3	2	3.5	1	1
304	70	1	3	2	5	1	0
283	59	2	3	2	11	1	0
317	69	1	2	2	5.5	0	0
238	81	1	2	2	9.3	0	0
274	68	2	2	2	5	1	0
329	57	2	2	2	6.7	0	0
240	35	2	2	2	4.3	1	1
258	73	2	2	2	7	0	0
224	80	2	2	2	4.5	1	0
128	72	1	2	2	3.7	1	0
246	69	1	2	2	5	0	0
323	66	1	2	2	6.5	0	0
307	61	2	2	2	4.5	0	0
213	41	1	2	2	6	0	0
313	49	1	2	2	8	0	0
296	69	1	2	2	5	0	1
249	61	1	2	2	8.8	0	1
295	65	1	2	2	7.5	0	0
207	52	2	2	2	2.8	0	0
126	66	1	2	2	3	1	0
223	62	1	2	2	4.5	1	0
305	62	1	2	2	5	0	0
294	53	1	2	2	10	0	0
298	62	1	2	2	6.2	0	0
320	75	2	2	2	3.5	1	0
199	53	2	2	2	3.3	1	0
264	67	2	2	2	6	0	0
232	65	1	2	2	6.8	1	1
195	59	2	2	2	3	0	0
173	68	1	2	2	6	0	0
177	60	1	2	2	3.6	0	0
107	53	2	2	2	3.5	0	0
113	72	1	2	2	9	1	0
118	61	1	2	2	2.8	0	0
125	59	2	2	2	4.5	0	0
139	53	1	2	2	2	1	1
109	61	1	2	2	4.5	1	1
215	62	2	2	2	2.3	0	0
236	64	1	2	2	3.5	0	0
288	58	1	2	2	2	0	0
261	79	1	2	2	6	0	0

171	63	1	2	2	1.5	0	0
285	43	2	2	2	3.5	1	0
111	60	1	2	2	4.5	0	0
175	64	1	2	2	3	0	0
326	86	2	2	2	4.5	0	0
247	37	1	2	2	3.7	1	0
151	77	2	2	2	2	1	0
231	69	1	2	2	3	0	0
272	70	2	1	2	5.5	0	0
507	44	2	4	2	6.5	0	0
442	37	1	3	2	5.5	1	0
653	47	1	3	2	4.8	1	0
595	61	1	3	2	2	0	0
619	78	1	2	2	6	1	1
635	89	2	2	2	7.5	1	0
639	55	2	2	2	4.5	0	0
425	60	1	2	2	7	1	1
675	62	1	2	2	5	1	1
685	71	1	2	2	5.5	1	1
412	75	1	2	2	2.5	1	0
456	75	1	2	2	4.5	1	0
618	75	1	2	2	2.9	1	0
583	70	2	2	2	5	0	0
478	49	2	2	2	5	1	0
479	80	1	2	2	3	1	0
614	59	1	2	2	7.2	1	1
419	40	1	2	2		1	0
422	82	1	2	2	9	1	0
458	54	1	2	2	5.5	1	0
660	74	1	2	2	6.5	1	0
451	63	1	2	2	2	0	1
535	47	2	2	2	3.4	0	0
616	54	2	2	2	5	0	1
648	52	1	2	2	5	0	0
485	81	2	2	2	5.6	0	0
431	76	1	2	2	1.4	1	0
435	67	1	2	2	2.5	1	0
474	33	1	2	2	5	1	0
477	74	2	2	2	7	1	1
584	56	2	2	2	7	1	0
641	61	1	2	2	4	1	1
446	29	1	2	2	5	1	0
523	51	1	2	2	6	1	0

596	74	1	2	2	6.5	1	0
629	61	1	2	2	4.8	1	0
661	74	2	2	2	2.2	1	1
462	80	1	2	2	5	0	1
513	72	1	2	2	6.5	0	0
657	64	1	2	2	4.4	0	1
407	68	1	2	2	6.2	0	0
411	67	1	2	2	3	0	0
418	62	1	2	2	3.5	0	0
426	73	1	2	2	3	0	0
467	76	1	2	2	3.8	0	0
488	58	2	2	2	2.3	0	0
502	61	2	2	2	7.5	0	0
510	70	2	2	2	5	0	0
514	57	2	2	2	4.5	0	0
516	77	2	2	2	5.1	0	0
539	61	1	2	2	6.5	0	0
549	58	1	2	2	5	0	0
578	67	2	2	2	5	0	0
580	45	1	2	2	6	0	0
597	81	2	2	2	4.2	0	0
613	29	2	2	2	1.7	0	0
672	71	2	2	2	4.3	0	0
691	72	2	2	2	2	0	0
464	66	1	2	2	2	1	0
481	52	1	2	2	5	1	1
482	56	2	2	2	4.5	1	1
455	64	1	2	2	4.3	1	1
503	62	1	2	2	2.2	1	0
554	48	2	2	2	8.5	1	1
577	72	1	2	2	5.5	1	0
483	66	1	2	2	3	0	0
498	73	1	2	2	4.5	0	0
509	61	1	2	2	3.7	0	0
620	74	1	2	2	4.5	0	1
624	60	1	2	2	2.8	0	0
676	61	1	2	2	5.6	0	0
438	65	1	2	2	6.4	0	0
480	77	2	2	2	3.5	0	1
504	49	1	2	2	3.5	0	0
508	45	1	2	2	6.5	0	0
515	58	2	2	2	3.5	0	0
522	71	2	2	2	5	0	0

556	75	2	2	2	7	0	0
558	52	2	2	2	2.1	0	0
591	54	1	2	2	6.5	0	0
599	84	2	2	2	5.3	0	0
603	77	1	2	2	6.5	0	0
610	67	1	2	2	5	0	0
644	73	1	2	2	4.5	0	0
659	88	2	2	2	3.7	0	0
670	70	1	2	2	5	0	0
517	81	2	2	2	3.3	1	0
403	35	2	2	2	4	0	0
427	74	2	2	2	2.2	0	0
440	63	1	2	2	3.5	0	0
528	71	2	2	2	2.7	0	0
559	80	1	2	2	1.9	0	0
683	57	1	2	2	2.1	0	0
465	60	1	2	2	1.7	0	0
538	44	2	2	2	1.7	0	0
561	71	2	2	2	3	0	0
469	35	2	1	2	3.3	0	0
537	50	1	1	2	5	1	0
663	90	2	1	2	8	0	0
432	50	2	2	2	6	1	0
668	80	1	2	2	9	1	0
592	65	1	1	2	8	1	0
550	68	1	1	2	6.5	0	0
505	47	1	1	2	3.2	0	0
601	66	2	1	2	3	0	0
679	65	1	1	2	3.2	0	0
560	73	2	1	2	3	0	0
677	85	1	1	2	2	0	0
184	59	1	3	1	3.5	1	0
119	48	1	3	1	10	1	0
164	67	2	3	1	9	1	1
214	74	2	3	1	10	1	1
314	58	2	3	1	10	0	0
138	52	1	3	1	6	1	1
161	70	2	3	1	8	1	1
278	60	2	2	1	4.5	1	0
135	66	1	2	1	10	1	0
150	71	1	2	1	6	1	0
309	56	2	2	1	5	0	1
291	59	1	2	1	3.5	1	0

108	60	1	2	1	6	1	0
102	72	2	2	1	6.5	0	0
260	75	2	2	1	4	0	1
162	64	1	2	1	5	1	0
282	80	2	2	1	10	0	0
284	65	2	2	1	11.6	0	0
105	56	2	2	1	5	1	0
193	63	1	2	1	3	1	0
325	56	1	2	1	3	1	0
190	54	2	2	1	7.5	0	0
100	83	1	2	1	6	1	0
312	65	1	2	1	6.5	1	1
147	50	2	2	1	5	0	0
322	58	2	2	1	4.5	1	0
156	74	1	2	1	7	0	0
112	55	1	2	1	9	0	0
252	57	1	2	1	4	0	0
174	58	2	2	1	5.5	0	0
166	71	2	2	1	2.5	1	0
110	64	2	2	1	3.5	0	0
122	62	1	2	1	5	1	0
218	60	2	2	1	1.6	0	0
192	62	2	2	1	3.7	0	0
234	61	1	2	1	3.5	0	0
140	63	1	2	1	3	0	0
276	68	1	2	1	3	1	0
191	64	2	1	1	3.5	1	0
328	60	1	1	1	2.5	0	0
182	63	1	3	1	3	0	0
281	82	2	2	1	6	0	0
286	48	2	2	1	4	0	0
98	55	2	2	1	9.6	0	0
15	78	1	2	1	2.3	1	0
302	62	1	1	1	3.5	0	0
423	64	2	4	1	5.5	1	0
565	48	1	4	1	8	1	0
674	79	1	4	1	6.4	0	1
564	69	1	4	1	6	0	0
590	57	2	3	1	2.5	1	0
526	38	1	3	1	5.5	1	0
680	86	2	3	1	5.5	0	0
612	65	2	3	1	5	1	1
415	80	2	3	1	10	1	0

527	36	1	3	1	7	1	0
689	81	2	3	1	6.1	1	0
443	36	2	3	1	7.5	0	0
506	60	1	3	1	7.5	0	0
632	27	1	3	1	11	0	0
623	53	1	3	1	2.7	1	0
449	80	2	3	1	10.5	0	0
690	84	2	3	1	3	0	0
473	38	1	3	1	6.5	1	0
637	66	1	2	1	5.5	1	1
673	71	2	2	1	5	0	0
401	69	2	2	1	4	1	0
606	73	2	2	1	5	1	0
567	55	2	2	1	4	1	0
604	71	1	2	1	4	1	1
569	71	2	2	1	5.7	0	0
671	64	1	2	1	2.5	1	0
555	59	2	2	1	4.5	0	0
649	61	1	2	1	5	0	0
470	32	2	2	1	6	0	0
533	79	1	2	1	9.3	0	0
542	56	2	2	1	1.6	1	0
631	68	1	2	1	6	1	0
402	59	2	2	1	9	1	0
428	87	2	2	1	6	1	0
452	77	2	2	1	4	1	0
540	43	1	2	1	5	1	0
647	52	2	2	1	5.5	0	0
500	77	2	2	1	7.1	0	0
573	75	1	2	1	4	0	0
611	60	2	2	1	6	0	0
651	54	2	2	1	3	0	0
582	57	1	2	1	5	1	0
536	77	1	2	1	5.9	1	0
643	39	2	2	1	5.8	1	0
662	73	1	2	1	5	1	0
688	79	2	2	1	2.2	1	0
531	74	2	2	1	5	0	0
551	62	1	2	1	8	0	1
489	66	1	2	1	2	0	0
658	62	2	2	1	2.7	1	0
421	60	1	2	1	4	1	0
491	80	1	2	1	6	1	0

622	85	2	2	1	5.5	1	0
636	66	1	2	1	6	1	0
408	79	2	2	1	3.2	0	0
490	77	1	2	1	7	0	0
557	50	1	2	1	6.6	0	0
563	31	2	2	1	6	0	0
626	74	2	2	1	4.5	0	0
628	71	2	2	1	6.5	0	0
630	74	2	2	1	4	0	0
633	55	2	2	1	5.3	0	0
638	80	2	2	1	4	0	0
681	76	1	2	1	5	0	0
686	85	2	2	1	8	0	0
587	52	2	2	1	0.9	1	0
605	62	1	2	1	1.5	1	0
575	76	1	2	1	2	1	0
414	70	1	2	1	2.6	0	0
429	72	2	2	1	4	0	0
453	73	2	2	1	2	0	0
645	49	1	2	1	1.8	0	0
667	67	1	2	1	2	0	0
600	66	1	4	1	8.8	0	0
448	75	2	4	1	1.6	0	0
684	74	1	1	1	2.5	1	0
501	57	1	1	1	7.5	0	0
678	74	2	1	1	5	0	0

PNI	ade_back	dz_death	dist_meta	recur	diseaseFre	lastFU_mo	dzfreeFU
0	0	0	0	0	0	12.9	12.9
1	0	0	0	0	0	38.3	38.3
0	0	0	0	0	0	45	45
1	0	0	0	0	0	60.2	60.2
0	0	0	0	0	0	60.2	60.2
0	0	1	0	0	0	5.7	5.7
0	0	0	0	0	0	94.8	94.8
0	0	0	0	0	0	60.2	60.2
0	0	1	1	0	1	5.1	5
0	0	1	1	1	1	19.4	5.5
0	0	0	1	0	1	79.6	7.1
1	0	1	1	0	1	23.1	7.3
0	0	1	1	0	1	39.7	27.4
0	0	0	0	0	0	43.1	43.1
1	0	1	1	1	1	34.8	34.2
0	0	1	0	0	0	0.1	0.1
0	0	0	0	0	0	0.4	0.4
0	0	1	0	0	0	0.7	0.7
0	0	0	0	0	0	1.4	1.4
0	0	0	0	0	0	2.4	2.4
1	0	1	0	0	0	4.2	4.2
0	0	0	0	0	0	5.8	5.8
0	0	0	0	0	0	7.2	7.2
1	0	0	0	0	0	8.5	8.5
0	0	0	0	0	0	12.1	12.1
0	0	0	0	0	0	12.8	12.8
1	0	0	0	0	0	51.1	51.1
0	0	0	0	0	0	60	60
0	0	0	0	0	0	60	60
0	0	0	0	0	0	60.2	60.2
0	0	0	0	0	0	60.2	60.2
0	0	0	0	0	0	60.3	60.3
1	0	0	0	0	0	60.4	60.4
1	0	0	0	0	0	60.5	60.5
0	0	0	0	0	0	60.8	60.8
0	0	0	0	0	0	60.9	60.9
0	0	0	0	0	0	61.1	61.1
1	0	0	0	0	0	61.1	61.1
0	0	0	0	0	0	62.5	62.5
0	0	0	0	0	0	66.1	66.1
0	0	0	0	0	0	67.8	67.8
0	0	0	0	0	0	68.9	68.9

0	0	0	0	0	0	73.6	73.6
1	0	0	0	0	0	73.8	73.8
0	0	0	0	0	0	75.3	75.3
0	0	0	0	0	0	75.9	75.9
0	0	0	0	0	0	76.5	76.5
0	0	0	0	0	0	77.9	77.9
0	0	0	0	0	0	78.7	78.7
0	0	0	0	0	0	80.2	80.2
0	0	0	0	0	0	80.6	80.6
0	0	0	0	0	0	81.3	81.3
0	0	0	0	0	0	82.9	82.9
0	0	0	0	0	0	84.7	84.7
0	0	0	0	0	0	85.8	85.8
0	0	0	0	0	0	91.5	91.5
0	0	0	0	0	0	92.1	92.1
0	0	0	0	0	0	92.5	92.5
0	0	0	0	0	0	94.7	94.7
1	0	0	0	0	0	96.3	96.3
0	0	0	0	0	0	98.5	98.5
0	0	0	1	0	1	50.8	9.3
1	0	1	1	0	1	33.7	14.7
0	0	0	1	0	1	76.6	18.6
1	0	0	0	1	1	92.9	19.8
1	0	1	1	1	1	38.3	29.7
1	0	0	1	0	1	31	30.6
0	0	0	1	0	1	47.5	43.9
1	0	1	1	0	1	54.4	51.7
0	0	0	1	1	1	82.1	58.1
0	0	0	1	0	1	81.5	71.4
0	0	0	0	0	0	1.5	1.5
0	0	0	0	0	0	1.5	1.5
0	0	0	0	0	0	12.2	12.2
0	0	0	0	0	0	37.6	37.6
0	0	0	0	0	0	39.7	39.7
0	0	0	0	0	0	60.2	60.2
0	0	0	0	0	0	60.2	60.2
0	0	0	0	0	0	60.4	60.4
0	0	0	0	0	0	60.4	60.4
0	0	0	0	0	0	60.6	60.6
0	0	0	0	0	0	60.7	60.7
0	0	0	0	0	0	60.9	60.9
0	0	0	0	0	0	61.5	61.5
0	0	0	0	0	0	62.2	62.2

1	0	0	0	0	0	73.6	73.6
0	0	0	0	0	0	80	80
0	0	0	0	0	0	89.1	89.1
0	0	0	1	0	1	83.4	60
0	0	0	0	0	0	58.6	58.6
0	0	0	0	0	0	59.9	59.9
0	0	0	0	0	0	60	60
0	0	0	0	0	0	60.6	60.6
1	0	0	0	0	0	7	7
0	0	0	0	0	0	60	60
1	1	0	0	0	0	59.5	59.5
0	1	0	0	0	0	96.7	96.7
0	1	0	0	0	0	6.1	6.1
0	1	0	0	0	0	59.8	59.8
0	1	0	0	0	0	60.2	60.2
0	1	0	0	0	0	61.3	61.3
0	1	0	0	0	0	64	64
0	1	0	0	0	0	19	19
1	1	0	0	0	0	60	60
0	1	0	0	0	0	28.7	28.7
0	1	0	0	0	0	47.8	47.8
1	0						
0	0						
1	0						
0	0						
0	0						
1	0						
1	0						
1	0						
0	0						
0	0						
1	0						
1	0						
1	0						
0	0						
0	0						
0	0						
0	0						
0	0						
1	0						
1	0						
1	0						
0	0						

0	0
1	0
1	0
1	0
1	0
0	0
0	0
0	0
0	0
0	0
0	0
0	0
1	0
1	0
1	0
0	0
0	0
0	0
0	0
0	0
0	0
0	0
1	0
1	0
1	0
0	0
0	0
1	0
1	0
1	0
0	0
0	0
0	0
0	0
0	0
0	0
0	0
0	0
0	0
0	0
0	0
0	0
0	0

0	0						
0	0						
0	0						
0	0						
0	0						
1	0						
0	0						
0	0						
0	0						
0	0						
0	0						
0	0						
0	0						
0	0						
0	0						
0	0						
0	0						
0	0						
0	0						
0	0						
0	0						
0	0						
0	0						
0	0						
0	0						
0	0						
0	0						
0	0						
0	0						
0	0						
0	0						
0	0						
0	0						
0	0						
0	0						
0	0						
0	1						
1	1						
0	1						
0	1						
0	1						
0	1						
1	0	0	1	0	1	66.7	7.2

1	0	1	1	0	1	28.3	8.6
1	0	1	1	0	1	23.5	6.1
0	0	1	1	0	1	19.5	6.6
1	0	0	1	0	1	76.2	14.4
0	0	0	0	0	0	25.8	25.8
0	0	0	0	0	0	39.3	39.3
0	0	0	0	0	0	55.6	55.6
0	0	0	0	0	0	70.3	70.3
1	0	1	1	0	1	29.5	11.7
0	0	0	0	0	0	12.4	12.4
0	0	0	0	0	0	12.4	12.4
1	0	0	0	0	0	39.4	39.4
0	0	0	0	0	0	51.4	51.4
1	0	0	0	0	0	54.8	54.8
0	0	0	0	0	0	60	60
0	0	0	0	0	0	60.2	60.2
0	0	0	0	0	0	60.2	60.2
0	0	0	0	0	0	60.2	60.2
0	0	0	0	0	0	60.3	60.3
0	0	0	0	0	0	60.3	60.3
1	0	0	0	0	0	60.4	60.4
1	0	0	0	0	0	60.4	60.4
0	0	0	0	0	0	60.4	60.4
0	0	0	0	0	0	60.5	60.5
0	0	0	0	0	0	60.6	60.6
0	0	0	0	0	0	62.4	62.4
0	0	0	0	0	0	62.7	62.7
0	0	0	0	0	0	65.9	65.9
0	0	0	0	0	0	73.4	73.4
1	0	0	0	0	0	80.7	80.7
0	0	0	0	0	0	90.2	90.2
0	0	0	0	0	0	90.4	90.4
0	0	0	0	0	0	92.6	92.6
1	0	0	0	0	0	94.1	94.1
0	0	0	0	0	0	97	97
0	0	0	0	0	0	97.1	97.1
1	0	0	0	0	0	97.4	97.4
1	0	1	1	0	1	48.7	8
0	0	1	1	0	1	38.6	11.7
1	0	1	1	0	1	40.7	12.9
0	0	0	1	0	1	99.3	12.9
1	0	0	0	1	1	40.9	36.1
0	0	0	1	0	1	51.5	37.6

0	0	0	0	0	0	59.9	59.9
0	0	0	0	0	0	60.5	60.5
0	0	0	0	0	0	67.7	67.7
0	0	0	0	0	0	84.3	84.3
0	1	0	0	0	0	0.9	0.9
0	1	0	0	0	0	60.3	60.3
0	1	0	0	0	0	38.5	38.5
0	1	0	0	0	0	88.2	88.2
0	1	0	0	0	0	61	61
0	0						
1	0						
1	0						
0	0						
1	0						
0	0						
1	0						
1	0						
1	0						
1	0						
1	0						
1	0						
0	0						
0	0						
1	0						
1	0						
1	0						
0	0						
0	0						
0	0						
0	0						
1	0						
1	0						
1	0						
1	0						
0	0						
1	0						
1	0						
1	0						
1	0						
1	0						
1	0						
0	0						
0	0						

0	0
0	0
0	0
1	0
1	0
1	0
0	0
0	0
0	0
0	0
0	0
0	0
0	0
0	0
0	0
0	0
0	0
0	0
0	0
0	0
0	0
0	0
0	0
0	0
1	0
1	0
1	0
0	0
0	0
0	0
0	0
1	0
1	0
1	0
1	0
1	0
1	0
0	0
0	0
0	0
0	0
0	0
0	0

0	0						
0	0						
0	0						
0	0						
0	0						
0	0						
0	0						
0	0						
0	0						
0	0						
0	0						
0	0						
0	0						
0	0						
0	0						
0	0						
0	0						
0	0						
0	0						
1	0						
0	0						
0	0						
1	1						
0	1						
1	1						
0	1						
0	1						
0	1						
0	1						
0	1						
0	1						
1	0	1	1	0	1	25	5.7
1	0	1	1	0	1	12.8	6.7
1	0	1	1	0	1	29	11.6
1	0	1	0	0	0	4.2	4.2
0	0	0	0	0	0	68.2	68.2
1	0	1	1	0	1	9.3	0.7
1	0	1	1	1	1	7.9	6.5
0	0	0	0	0	0	60.2	60.2
0	0	0	0	0	0	60.2	60.2
0	0	1	1	0	1	30.6	11.6
1	0	0	1	0	1	62.7	24.3
1	0	0	1	0	1	42.7	42.3

0	0	0	0	0	0	0.6	0.6
0	0	1	0	0	0	1.3	1.3
0	0	0	0	0	0	1.5	1.5
0	0	0	0	0	0	5.7	5.7
0	0	0	0	0	0	5.8	5.8
0	0	1	0	0	0	6.3	6.3
0	0	0	0	0	0	40	40
1	0	0	0	0	0	50	50
0	0	0	0	0	0	60.4	60.4
0	0	0	0	0	0	60.4	60.4
0	0	0	0	0	0	68.3	68.3
0	0	0	0	0	0	68.7	68.7
0	0	0	0	0	0	72.1	72.1
0	0	0	0	0	0	74.1	74.1
0	0	0	0	0	0	81.9	81.9
0	0	0	0	0	0	82.6	82.6
0	0	0	0	0	0	85.9	85.9
0	0	0	0	0	0	89.9	89.9
0	0	0	0	0	0	91	91
0	0	0	0	0	0	94.1	94.1
0	0	0	1	0	1	11.9	78
0	0	0	0	0	0	9.6	9.6
1	0	0	0	0	0	66.9	66.9
0	0	0	0	0	0	83	83
0	0	0	0	0	0	94.9	94.9
0	0	0	0	0	0	0.6	0.6
0	0	0	0	0	0	31.6	31.6
0	0	0	0	0	0	60.2	60.2
0	1	0	0	0	0	55.5	55.5
1	1	0	0	0	0	33.3	33.3
0	1	0	0	0	0	60.3	60.3
0	1	0	0	0	0	71	71
0	1	0	0	0	0	6.7	6.7
0	1	0	0	0	0	58	58
1	0						
1	0						
0	0						
0	0						
1	0						
0	0						
0	0						
1	0						
0	0						

0	0
0	0
0	0
0	0
0	0
0	0
0	0
0	0
0	0
0	0
0	0
1	0
1	0
0	0
0	0
1	0
1	0
1	0
1	0
0	0
0	0
1	0
1	0
0	0
0	0
0	0
0	0
1	0
0	0
0	0
0	0
0	0
1	0
0	0
0	0
0	0
0	0
1	0
1	0
0	0
1	0
0	0
0	0

0	0
0	0
0	0
0	0
0	0
0	0
0	0
0	0
0	0
0	0
0	0
0	0
0	0
0	0
0	0
0	0
0	0
0	0
0	0
0	0
0	0
0	1
0	1
0	1
0	1
0	1

meta_Initia	pT	pN_0_1_2	CTx	LVI
0	3	0	0	0
0	3	0	3	0
0	3	0	3	0
0	3	0	3	0
0	3	0	0	0
0	4	2	0	1
0	4	1	1	1
0	3	1	3	1
0	3	1	3	1
0	3	0	0	1
0	3	2	3	1
0	3	2	3	1
0	3	2	3	1
0	4	2	3	1
0	4	2	1	0
0	3	2	0	1
0	3	2	0	0
0	3	1	0	0
0	3	2	0	1
0	3	0	0	0
0	3	2	0	1
0	3	0	3	0
0	3	1	0	0
0	3	1	3	1
0	3	0	3	0
0	3	0	3	0
0	3	0	3	0
0	3	2	3	1
0	3	1	3	1
0	3	0	3	1
0	3	0	3	0
0	3	0	3	1
0	3	1	3	0
0	3	1	3	0
0	3	0	3	0
0	3	0	0	0
0	3	1	3	1
0	3	0	3	1
0	3	2	3	1
0	3	0	3	1
0	3	0	3	0
0	3	1	3	1

0	3	0	0	1
0	3	1	3	0
0	3	0	3	1
0	3	0	3	1
0	3	0	3	0
0	3	0	3	0
0	3	0	3	0
0	3	0	3	0
0	3	0	3	1
0	3	1	3	0
0	3	2	1	1
0	3	0	3	0
0	3	0	3	0
0	3	0	3	1
0	3	0	3	0
0	3	0	0	0
0	3	1	1	1
0	3	0	3	1
0	3	1	2	0
0	3	1	3	0
0	3	1	1	1
0	3	0	3	0
0	3	2	1	1
0	3	2	3	0
0	3	2	0	0
0	3	1	0	0
0	3	2	3	1
0	3	2	3	1
0	3	0	3	0
0	2	2	0	0
0	2	0	3	1
0	2	0	0	0
0	2	1	3	0
0	2	0	3	0
0	2	1	3	1
0	2	0	3	0
0	2	1	3	0
0	2	0	3	1
0	2	0	3	0
0	2	0	3	0
0	2	0	3	0
0	2	0	3	0
0	2	0	3	0

0	2	1	3	0
0	2	1	3	1
0	2	0	3	0
0	2	1	3	1
0	1	0	0	0
0	1	0	0	0
0	1	0	0	0
0	1	0	0	0
0	3	0	3	0
0	3	2	0	1
0	3	1	3	0
0	3	0	3	1
0	2	0	3	0
0	1	0	0	0
0	1	0	0	0
0	1	1	3	1
0	1	0	0	0
0	3	0	3	0
0	2	0	3	0
0	1	0	0	0
0	1	0	0	0
	3	1		1
	3	1		0
	3	0		1
	3	0		1
	3	0		0
	4	2		1
	4	2		1
	4	1		0
	4	1		0
	4	0		0
	3	2		1
	3	2		1
	3	2		1
	3	2		1
	3	2		1
	3	2		1
	3	2		1
	3	2		1
	3	2		0
	3	2		0
	3	2		1
	3	2		0

3	2	0
3	1	1
3	1	1
3	1	1
3	1	1
3	1	1
3	1	1
3	1	1
3	1	1
3	1	1
3	1	1
3	1	1
3	1	0
3	1	0
3	1	0
3	1	0
3	1	0
3	1	0
3	1	0
3	1	0
3	1	0
3	1	0
3	0	1
3	0	1
3	0	1
3	0	1
3	0	1
3	0	0
3	0	0
3	0	0
3	0	0
3	0	0
3	0	0
3	0	0
3	0	0
3	0	0
3	0	0
3	0	1
3	0	0
3	0	0
3	0	0
3	0	0
3	0	0

	3	0		0
	3	0		0
	3	0		0
	3	0		0
	3	0		0
	2	1		1
	2	1		1
	2	1		1
	2	1		0
	2	1		0
	2	1		0
	2	0		1
	2	0		1
	2	0		0
	2	0		0
	2	0		0
	2	0		0
	2	0		0
	2	0		0
	2	0		0
	2	0		0
	1	1		1
	1	1		1
	1	1		0
	1	1		0
	1	0		1
	1	0		0
	1	0		0
	1	0		1
	1	0		0
	1	0		0
	1	0		0
	3	1		0
	3	1		0
	3	0		0
	2	1		1
	2	1		1
	2	0		1
	2	0		0
	1	1		1
	1	0		0
	1	0		0
0	3	1	1	0

0	4	1	1	1
0	3	0	3	1
0	3	2	3	1
0	3	2	1	1
0	4	1	3	0
0	4	0	3	0
0	4	2	1	1
0	4	0	1	0
0	4	2	1	1
0	3	1	3	0
0	3	0	0	1
0	3	2	3	1
0	3	0	3	0
0	3	0	3	0
0	3	1	1	0
0	3	2	1	0
0	3	1	1	0
0	3	0	3	1
0	3	0	3	1
0	3	0	3	0
0	3	2	1	0
0	3	0	3	1
0	3	0	3	1
0	3	1	1	0
0	3	0	3	0
0	3	0	3	0
0	3	1	3	1
0	3	2	1	1
0	3	0	3	0
0	3	1	1	1
0	3	2	1	0
0	3	1	1	0
0	3	0	3	0
0	3	1	1	0
0	3	1	1	1
0	3	0	3	0
0	3	1	1	0
0	3	2	2	1
0	3	1	3	1
0	3	1	1	0
0	3	0	3	0
0	3	1	3	0
0	3	0	3	0

0	2	1	1	0
0	2	1	1	1
0	2	0	3	0
0	2	0	3	0
0	3	0	0	0
0	2	0	3	1
0	1	0	0	1
0	1	0	0	0
0	3	1	3	0
	4	1		0
	3	1		1
	3	1		1
	2	0		0
	4	2		1
	4	2		1
	4	2		0
	4	1		1
	4	1		1
	4	1		1
	4	0		1
	4	0		1
	4	0		1
	4	0		0
	3	2		1
	3	2		1
	3	2		1
	3	2		1
	3	2		1
	3	2		1
	3	2		1
	3	2		1
	3	2		0
	3	2		1
	3	2		0
	3	2		0
	3	1		1
	3	1		1
	3	1		1
	3	1		1
	3	1		1
	3	1		1
	3	1		1
	3	1		1

3	1	1
3	1	1
3	1	1
3	1	1
3	1	0
3	1	1
3	1	0
3	1	0
3	1	0
3	1	0
3	1	0
3	1	0
3	1	0
3	1	0
3	1	0
3	1	0
3	1	0
3	1	0
3	1	0
3	1	0
3	1	0
3	1	0
3	1	0
3	1	0
3	0	1
3	0	1
3	0	1
3	0	1
3	0	1
3	0	1
3	0	1
3	0	0
3	0	0
3	0	0
3	0	1
3	0	0
3	0	0
3	0	0
3	0	1
3	0	0
3	0	0
3	0	0
3	0	0

	3	0		0
	3	0		0
	3	0		0
	3	0		0
	3	0		0
	3	0		0
	3	0		0
	3	0		0
	3	0		0
	2	0		1
	2	0		0
	2	0		0
	2	0		0
	2	0		0
	2	0		0
	2	0		0
	1	0		0
	1	0		0
	1	0		0
	3	1		0
	3	0		1
	3	0		0
	4	1		1
	3	0		1
	3	0		1
	3	0		0
	2	0		0
	2	0		0
	2	0		0
	1	0		0
	1	0		0
0	4	0	3	1
0	4	0	0	1
0	4	2	1	1
0	3	2	3	1
0	3	0	3	0
0	3	1	1	1
0	3	2	3	1
0	4	1	1	1
0	4	0	1	1
0	4	2	3	1
0	4	2	1	1
0	4	2	1	1

0	3	0	0	1
0	3	0	0	0
0	3	0	3	1
0	3	2	3	1
0	3	1	3	0
0	3	0	3	0
0	3	1	1	1
0	3	1	1	1
0	3	0	3	1
0	3	0	3	0
0	3	0	0	1
0	3	2	1	1
0	3	1	1	0
0	3	1	1	1
0	3	0	3	0
0	3	0	3	0
0	3	0	3	0
0	3	1	1	0
0	3	1	1	1
0	3	0	3	0
0	3	0	3	1
0	2	0	3	0
0	2	0	3	0
0	2	0	3	0
0	2	0	3	0
0	1	0	0	1
0	3	1	1	1
0	3	0	3	0
0	3	2	1	0
0	3	0	0	0
0	3	0	3	0
0	3	2	1	0
0	1	0	3	1
0	2	0	3	0
	4	2		1
	4	2		1
	3	1		1
	3	0		0
	4	2		1
	4	2		1
	4	1		0
	3	2		1
	3	2		1

3	1	1
3	1	1
3	1	0
3	1	0
3	1	0
3	0	1
3	0	0
3	0	0
1	1	1
4	2	1
4	2	0
4	1	1
4	1	1
4	1	1
4	1	1
4	1	0
4	0	1
4	0	0
4	0	0
4	0	0
4	0	0
3	2	1
3	2	1
3	2	1
3	2	1
3	2	1
3	2	1
3	2	0
3	2	0
3	2	0
3	2	0
3	2	0
3	1	1
3	1	1
3	1	1
3	1	1
3	1	1
3	1	0
3	1	1
3	1	0
3	0	1
3	0	1
3	0	1

3	0	1
3	0	1
3	0	0
3	0	0
3	0	0
3	0	0
3	0	0
3	0	0
3	0	0
3	0	0
3	0	0
3	0	0
3	0	0
2	1	1
2	1	1
2	0	1
2	0	0
2	0	0
1	0	0
1	0	0
1	0	0
3	1	0
1	1	0
3	1	1
3	0	0
1	0	0
